# The significant association between quick return and depressive symptoms and sleep disturbances in paid workers: A nationwide survey

**DOI:** 10.3389/fpubh.2022.990276

**Published:** 2022-10-10

**Authors:** Byungyoon Yun, Juho Sim, Juyeon Oh, Yangwook Kim, Jin-Ha Yoon

**Affiliations:** ^1^Department of Preventive Medicine, Yonsei University College of Medicine, Seoul, South Korea; ^2^Department of Public Health, Graduate School, Yonsei University, Seoul, South Korea; ^3^The Institute for Occupational Health, Yonsei University College of Medicine, Seoul, South Korea

**Keywords:** quick return, depressive symptoms, sleep disturbances, paid workers, mediation

## Abstract

**Objectives:**

Although many studies have examined the association between shift work and depression or insomnia, few studies have examined the relationship between quick return (QR) to work and depressive symptoms, regardless of shift work. Thus, in this study, we aimed to assess the association between depressive symptoms (DS)/sleep disturbances (SDs) and QR.

**Methods:**

Data from the 6^th^ Korean Working Conditions Survey (2020) were used for this study. Paid workers aged between 20 and 65 years were included. DS were defined using the World Health Organization Well-Being Index (WHO-5) with a cut-off 50, and SD was defined as the occurrence of the following symptoms several times per month: difficulty in falling asleep, waking up in the middle of the night, or feeling tired even after waking up. QR was defined as “at least one case where the working interval between leaving work and the next day's work was < 11 h in the past month.” Multivariable logistic regression was performed to estimate the adjusted odd ratios (aORs) and 95% confidence intervals (CIs). Mediation analysis was conducted to examine whether SD was a significant mediator in the association between QR and DS.

**Results:**

Among the 27,554 participants, DS occurred in 8,277 patients, while SD occurred in 6,264 patients. The aORs (95% CIs) of DS and SD by QR were 2.01 (1.78–2.27) and 3.24 (2.87–3.66), respectively, after adjusting for age, gender, income, education, working hours, job status, working duration, region, shift work, and occupation. SD was a significant mediator in the association between QR and DS.

**Conclusion:**

QR is significantly associated with DS or SD regardless of demographic factors and the working environment. The significant relationship between QR and DS may be mediated by SD.

## Introduction

Depression, also known as major depressive disorder, is a widespread and notable medical ailment that can negatively affect one's feelings, thoughts, and behaviors. Depression symptoms include impaired attention, feelings of overwhelming guilt or low self-worth, despair about the future, thoughts of death or suicide, interrupted sleep, food- or weight-related changes, and feelings of extreme weariness or lack of energy (1).

As a debilitating disorder, depression can negatively affect people and their relationships with family and friends, work, sleeping and eating habits, and overall health (2). In 2019, depressive disorder prevalence worldwide was 5.02% for those aged 20 years or older and 4.75% for those aged between 20 and 54 years (3).

Before the coronavirus pandemic in South Korea (henceforth Korea), 19.8% of general health checkups in 2019 involved individuals with depressive symptoms (DS) (4), and the 2018 National Health and Nutrition Examination Survey showed that 21.0% of individuals were sufferers of DS (5). In Korea, Shin et al. (6) showed that only 4.17% of patients receive treatment for depression and that the estimated cost of such treatment was 3,461 USD per patient. Furthermore, depression is a contributing factor for both absenteeism and reduction in worker productivity (7, 8). Due to Coronavirus disease 2019 (COVID-19), the prevalence of depressive symptoms in Korea was 30.7% by strong association with changes in sleep pattern (9).

The prevalence of depressive symptoms or depression was considerably high in various occupational fields and nations. Approximately 20% of American social workers have been found to be on antidepressant medications, and 60% were found to have experienced depression in the past (10). Moreover, according to the multi-national meta-analysis including Asia countries reported that 28.8% of physicians were found to have suffered from depression (11). In addition, Western countries with similar role stress for nurses showed similar results (12, 13). The prevalence of depressive symptoms in Korean workers was also estimated by 39% based on the WHO-5 well-being index, with the highest prevalence of depressive symptoms in economic sector (14). Further, 21.0% of industrial workers were found to suffer from depression with similar estimated prevalence across the continents (22% for Asia, 18% for Europe, 20% for America)—a higher prevalence than that found among the general population (15). As such, many workers worldwide have complained about suffering from depression.

Contributing factors for depression among workers included sex, marital status, religious beliefs (16), disease (17, 18), diet, less leisure time (18, 19), shift work (20), long working hours, and lower salary (18). A study on depression among Korean workers found that depression was related to work stress (long working hours, making influence decisions, and work pressure) (21); furthermore, beyond work stress, job insecurity was also found to have an impact on depression (22).

Besides these factors, insomnia has been shown to be an important predictor of depression (23). Previous studies found that the improvement in insomnia is significantly associated with a decreased risk of depression (24, 25). Moreover, since quick return (QR) had significant association with higher risk of sleeplessness, sick leave, and injury in the previous studies (26–29), a study regarding the relationship or mediating effect between QR, insomnia, and depression might be needed. Furthermore, although many studies have examined the relationship between shift work disorder and depression, few studies have examined the relationship between DS and QR regardless of shift work.

Therefore, this study aims to investigate the relationship between DS/sleep disturbances (SDs) and QR independent of demographic and working conditions. Moreover, we aimed to establish the association between DS and QR while considering any indirect effects of SD.

## Methods

### Data sources and study population

This cross-sectional study utilized data from the Korean Working Conditions Survey [Sixth Korean Working Conditions Survey (KWCS), 2020], which has been conducted by the Occupational Safety and Health Research Institute since 2006. The study, which utilized questionnaires based on the European Working Conditions Survey, interviewed workers aged ≥ 15 years, who formed the study sample (30). The study participants were chosen randomly to represent the working population in Korea. The questionnaires included items regarding occupational classification, type of work, employment status, hazardous exposures, working profiles including working hours, QR, night shifts, and mental health effects of the participants.

Among 50,538 participants, 33,063 paid workers were initially enrolled in this study. Participants aged < 20 or > 65 years (*n* = 3,556) and those with missing values (*n* = 1,953) were excluded. The missing values included those related to working hours (*n* = 200), working years (*n* = 269), shift work (*n* = 81), QR (*n* = 150), SD (*n* = 19), DS (*n* = 42), income (*n* = 1,177), and education (*n* = 15).

This study was conducted according to the ethical requirements of the 1975 Declaration of Helsinki and was approved by the Institutional review board of Severance Hospital of the Yonsei University Health System (IRB No. 4-2021-1283). Informed consent was waived owing to the retrospective nature of this study.

### Variables and outcomes

This study's primary outcomes involved DS. The depressive group was defined using the World Health Organization Well-Being Index (WHO-5), which is used worldwide for assessing subjective well-being (31) (cut-off score: < 50) (32). The QR group was defined as participants who responded “yes” to the questionnaire item, “Has there been at least one case in the past month where the interval between leaving work and the next day's work was < 11 h?” The secondary outcome of this study involved SD. The questionnaires defined SD as the presence of any of the following symptoms at least several times per month: difficulty in falling asleep, waking up in the middle of the night, and feeling tired even after waking up.

Age and gender were used based on the information of conducted survey and age was utilized as a continuous variable. Education level was classified into three groups based on participants' responses: high school graduate or under, college graduate, and university graduate. Income level was classified into three groups according to income per month: < 2 million KRW, 2 to < 4 million KRW, and ≥ 4 million KRW. Working hours were estimated for each week and stratified into three groups: ≤ 40 h, 41–51 h, and ≥ 52 h. Working duration was stratified into five groups: none, < 5 years, 5 to < 10 years, 10 to < 20 years, and ≥ 20 years. Employment type was classified into three groups: full-time, part-time, and temporary workers. Occupational classification was stratified into three groups: white collar (managers, professionals and related workers, and clerks), blue collar (craft and related trades workers, equipment workers, machine operating and assembling workers, and elementary workers), and others (service workers, sales workers, and skilled agricultural, forestry, and fishery workers). Shift work was defined as “yes” or “no” based on the response of the related question “I do shift work.” Central and rural regions were defined based on the residential area of the participants.

### Statistical analyses

The baseline characteristics of the participants stratified according to QR were compared using chi-squared test and independent *t*-test for categorical and continuous variables, respectively. Multivariable logistic regression modeling was sequentially performed in order to estimate the adjusted odds ratios (ORs) and confidence intervals (CIs) of DS and SD based on QR. Model 1 was adjusted by age and sex. Model 2 was adjusted by age, sex, income, and education level. Finally, model 3 was adjusted by working hours, working duration, employment type, occupational classification, region, and shift work in addition to covariates used in model 2. Subgroup analysis stratified by gender was further conducted along with multivariable logistic regression models. Moreover, interaction analysis was implemented in order to examine the interactions between long working hours and QR. Furthermore, mediation analysis was conducted in order to examine whether SD were a significant mediator in associating QR with DS. Mediation analysis was performed according to the Baron and Kenny method, and the Sobel test was performed (33).

Regarding sensitivity analyses, propensity score matching (PSM) (ratio: 1:3) was performed using the nearest neighbor method (caliper width: 0.1); the matching variables were age, gender, income, education level, working hours, working duration, employment type, occupational classification, region, and shift work. The absolute mean standardized difference was calculated for each variable to evaluate the PSM balance. The baseline characteristics of the participants, which were stratified using QR in the matched cohort, were compared using a chi-squared test and an independent *t*-test. Furthermore, multivariable logistic regression modeling was conducted, and the different cut-off of WHO-5 index score (≤ 28) was applied for depression as a sensitivity analysis (34, 35). Adjusted ORs (95% CIs) of depression were estimated for workers with a working duration encompassing more than 1 year.

All statistical analyses were two-sided, and *p* < 0.05 was considered significant. This study used R software version 4.0.5 (R Foundation for Statistical Computing, Vienna, Austria) to perform all statistical analyses.

## Results

### Baseline characteristics of the participants

Among the 27,554 participants, the mean (standard deviation) age was 43.7 years (11.7), and 77.3% of the participants were men. Depression occurred in 8,277 participants (30.0%) [7,731 [29.3%] in the non-QR group and 546 [45.2%] in the QR group]. The baseline characteristics of the participants were stratified using QR, as summarized in Table 1. The QR group had a significantly higher prevalence among workers who had the following characteristics: young age, male, full-time, long working hours, long working duration, SD, high education level, high income level, central region, shift work, and depression (compared to the non-QR group [all *p* < 0.05]). Moreover, regarding the prevalence of DS and SD stratified by sex, women group has a significantly higher prevalence of DS and SD compared to men group [1983 [31.8%] vs. 6294 [29.5%] for DS, 1519 [24.3%] vs. 4745 [22.3%] for SD, respectively, all *p* < 0.001].

**Table 1:** Baseline characteristics of the participants (*n* = 27,554) in 6^th^ KWCS (2020) stratified by quick return.

**Variable**	**No quick return** **(*n* = 26,345)**	**Quick return** **(*n* = 1,209)**	***p*-value**
**Age**			0.01
Mean (SD)	43.68 (11.70)	42.87 (10.66)	
**Gender**			< 0.001
Women	6,039 (22.9%)	203 (16.8%)	
Men	20,306 (77.1%)	1,006 (83.2%)	
**Job status**			< 0.001
Full-time	21,589 (81.9%)	1,075 (88.9%)	
Part-time	3,411 (13.0%)	100 (8.3%)	
Temporary	1,345 (5.1%)	34 (2.8%)	
**Working hours**			< 0.001
≤ 40	18,887 (71.7%)	536 (44.3%)	
41–51	5,260 (20.0%)	370 (30.6%)	
≥52	2,198 (8.3%)	303 (25.1%)	
**Working duration**			< 0.001
< 1	4,274 (16.2%)	137 (11.3%)	
1 to < 5	9,995 (37.9%)	393 (32.5%)	
5 to < 10	5,629 (21.4%)	309 (25.6%)	
10 to < 15	4,449 (16.9%)	262 (21.7%)	
≥15	1,998 (7.6%)	108 (8.9%)	
**Sleep disturbance**			< 0.001
No	20,676 (78.5%)	614 (50.8%)	
Yes	5,669 (21.5%)	595 (49.2%)	
**Education**			< 0.001
High school graduate or under	10,889 (41.3%)	431 (35.6%)	
2-year college	5,254 (20.0%)	199 (16.5%)	
Over university	10,202 (38.7%)	579 (47.9%)	
**Income (10,000 KRW)**			< 0.001
< 200	7,320 (27.8%)	158 (13.1%)	
200 to < 400	15,110 (57.4%)	751 (62.1%)	
≥400	3,915 (14.8%)	300 (24.8%)	
**Region**			< 0.001
Rural	12,994 (49.3%)	554 (45.8%)	
Central	13,351 (50.7%)	655 (54.2%)	
**Occupation**			< 0.001
White collar	12,419 (47.2%)	595 (49.2%)	
Blue collar	7,331 (27.8%)	411 (34.0%)	
Others	6,595 (25.0%)	203 (16.8%)	
**Shift work**			< 0.001
No	23,994 (91.1%)	847 (70.1%)	
Yes	2,351 (8.9%)	362 (29.9%)	
**Depressive symptoms**			< 0.001
No	18,614 (70.7%)	663 (54.8%)	
Yes	7,731 (29.3%)	546 (45.2%)	

KRW, Korean won; KWCS, Korean Working Conditions Survey; SD, standard deviation.

### Relationships among QR, DS, and SD

**Figure 1:**
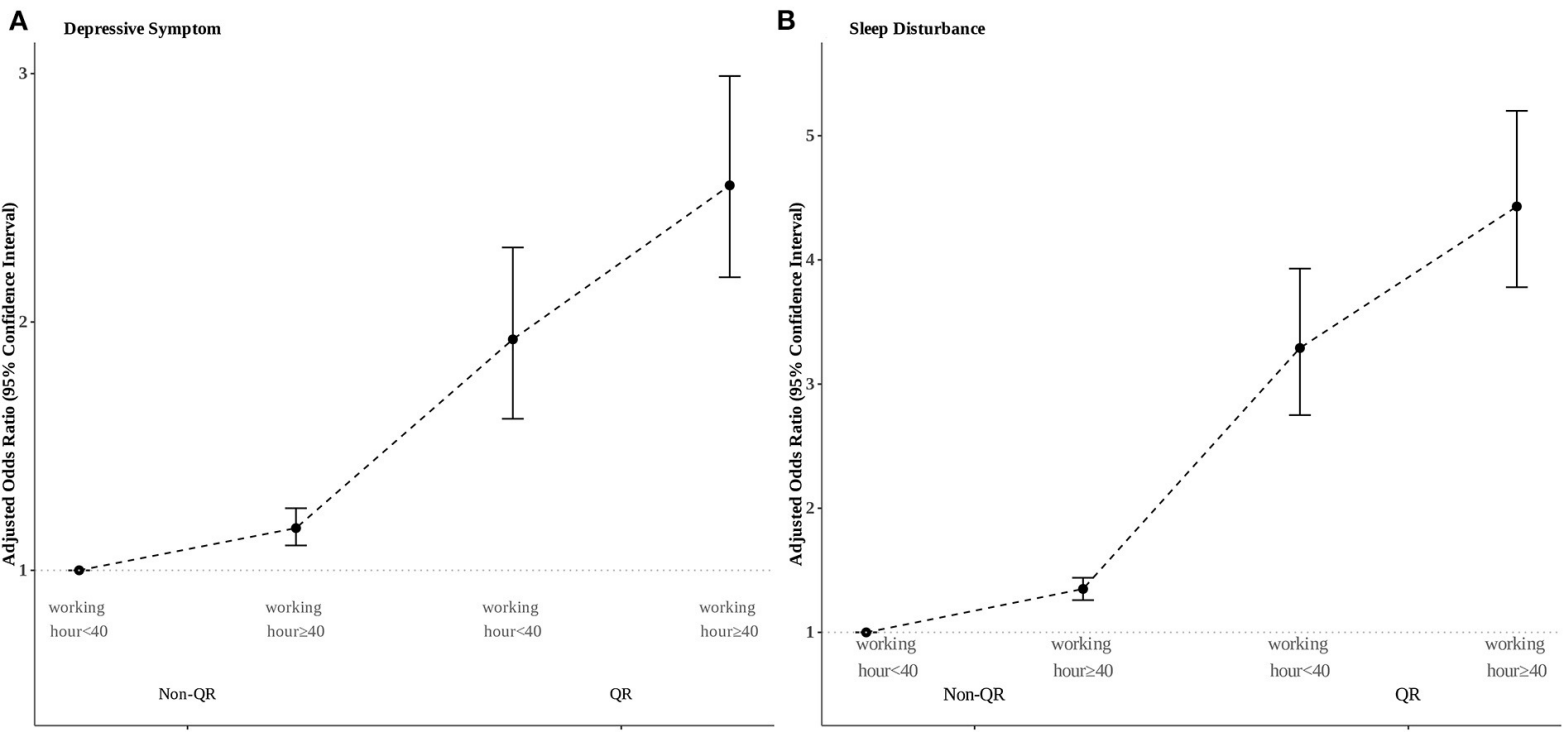
Interaction analysis of long working hours and quick return on **(A)** depressive symptoms and **(B)** sleep disturbance.

**Figure 2:**
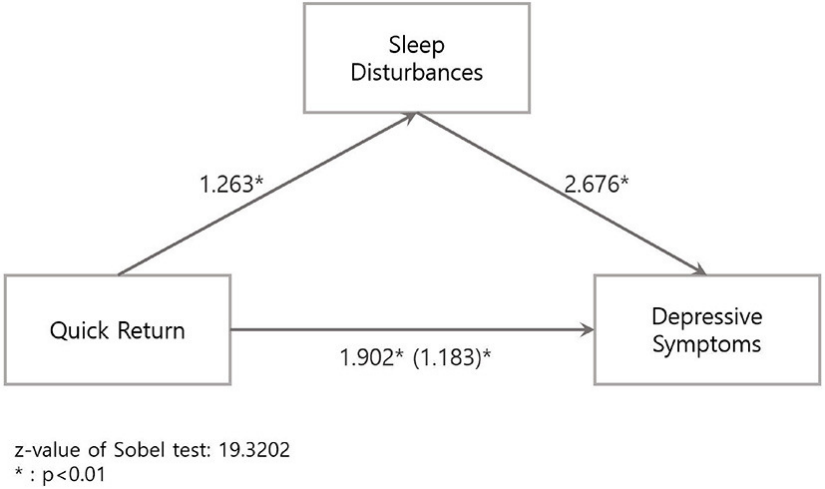
Mediation analysis of sleep disturbances on the association between quick return and depressive symptoms.

The association of QR with DS and SD was significant in the univariable logistic regression with crude ORs (95% CIs) of 1.98 (1.77–2.23) and 3.53 (3.15–3.97), respectively. After adjusting the covariates in the multivariable logistic regression models, the adjusted ORs (95% CI) of DS and SD by QR were 2.01 (1.78–2.27) and 3.24 (2.87–3.66), respectively, in the final model (Table 2). In the stratification analysis, the adjusted ORs (95% CIs) of DS and SD by QR in the final model were 2.12 (1.86–2.43) and 3.39 (2.96–3.87), respectively in men and 1.58 (1.18–2.12) and 2.62 (1.96–3.51), respectively in women; all of these were significant (Table 2). The interaction analysis results for QR, working hours, and depression are shown in Figure 1. Adjusted ORs (95% CIs) of DS and SD based on “QR & working hours ≥40 group,” “QR & working hours < 40 group,” and “non-QR & working hours ≥40 group” were 2.55 (2.18–2.99), 1.93 (1.61–2.30), and 1.17 (1.10–1.25), respectively for DS (Figure 1A) and 4.43 (3.78–5.20), 3.29 (2.75–3.93), and 1.35 (1.26–1.44), respectively for SD (Figure 1B), compared to the “non-QR & working hours < 40 group.” When mediation analysis was performed with SD as a mediator between QR and DS, because the direct effect (1.183) was less than the total effect (1.902), SD was found to be a partial mediation with partial interaction effects (z value of Sobel test: 19.3202) (Figure 2).

**Table 2:** Adjusted ORs (95% CIs) of depressive symptoms and sleep disturbance by quick return with stratification of gender in a logistic regression model.

	**Gender**	**Quick return**	**Crude model**	**Model 1**	**Model 2**	**Final model**
Depressive symptoms	Total	No	1.00 (reference)	1.00 (reference)	1.00 (reference)	1.00 (reference)
		Yes	1.98 (1.77–2.23)	2.04 (1.81–2.29)	2.17 (1.93–2.44)	2.01 (1.78–2.27)
	Male	No	1.00 (reference)	1.00 (reference)	1.00 (reference)	1.00 (reference)
		Yes	2.11 (1.86–2.39)	2.15 (1.89–2.44)	2.27 (2.00–2.59)	2.12 (1.86–2.43)
	Female	No	1.00 (reference)	1.00 (reference)	1.00 (reference)	1.00 (reference)
		Yes	1.54 (1.16–2.05)	1.57 (1.18–2.09)	1.72 (1.29–2.30)	1.58 (1.18–2.12)
Sleep disturbances	Total	No	1.00 (reference)	1.00 (reference)	1.00 (reference)	1.00 (reference)
		Yes	3.53 (3.15–3.97)	3.67 (3.26–4.13)	3.70 (3.28–4.16)	3.24 (2.87–3.66)
	Male	No	1.00 (reference)	1.00 (reference)	1.00 (reference)	1.00 (reference)
		Yes	3.71 (3.26–4.21)	3.80 (3.34–4.32)	3.86 (3.39–4.39)	3.39 (2.96–3.87)
	Female	No	1.00 (reference)	1.00 (reference)	1.00 (reference)	1.00 (reference)
		Yes	2.97 (2.24–3.94)	3.09 (2.33–4.10)	3.00 (2.25–3.98)	2.62 (1.96–3.51)

Model 1: adjusted by age.

Model 2: adjusted by age, income, and education.

Final model: adjusted by age, gender, income, education, working hours, sleep disturbance, job status, working duration, region, shift work, and occupation, except for each stratification variable.

OR, odds ratio; CI, confidence interval.

### Sensitivity analyses

After propensity matching (ratio: 1:3), 4,790 patients remained in the analysis. The baseline characteristics between the QR and non-QR group were well-balanced according to the absolute standardized mean difference below 0.1 (Supplementary Table S1). Multivariable logistic regression model analysis showed that the association of DS with QR was statistically significant [final model; adjusted OR [95% CI] 1.97 [1.72–2.25]]. Moreover, after using different cut-off scores ≤ 28 in the WHO-5 index, the result found a significant association with QR and DS [adjusted OR [95% CI] 2.25 [1.94–2.62] in the final model]. Additionally, analysis involving participants who worked for more than 1 year showed significant results [adjusted OR [95% CI] 1.99 [1.75–2.27] in the final model]. All the results related to sensitivity analyses are summarized in Supplementary Table S2.

## Discussion

This study used nationwide survey data to examine the significant association between QR and DS and SD. A multivariable logistic regression model analysis with adjustments of age, gender, income, education, working hours, SD, job status, working duration, region, shift work, and occupation confirmed the significant association of DS and SD with QR. Stratification analysis showed more predominant associations between depression and QR in men. Moreover, interaction analysis involving working hours showed a synergetic interaction between long working hours and QR regarding DS and SD. The mediation analysis demonstrated that SD was a mediator in the association between QR and DS. Finally, sensitivity analyses with PSM, using different cut-offs for the WHO-5 index score, and analysis of workers with working duration ≥ 1 year presented similar significant results.

Previous studies on QR and mental disorders (including insomnia, anxiety, and depression) mainly focused on individuals who performed shift work, especially health care workers such as nurses (27, 36, 37). These past studies presented insignificant associations between depression and QR and significant associations between sleep problems and QR. Unlike these past studies, our study found that QR was significantly related to both DS and SD. This result can be attributed to several reasons. First, our study included various occupations (not only health care workers) and identified a significant relationship between QR and DS/SD regardless of shift work and other working profiles. The effect of shift work may be underestimated due to the healthy shift worker effect, which involves a selection of shift work based on pre-employment screening and other health problems (38); it implies the underestimation of the effect of QR on the outcomes in the previous studies. Indeed, our study results showed that shift work was not significantly associated with DS (Supplementary Table S3) but significantly associated with SD. Further studies should consider the healthy worker effect, which this study could not clarify due to its limited retrospective cross-sectional design. Second, unlike previous studies, our study recruited a large number of participants. Because of the sufficient study sample size, our results may have sufficient statistical power.

In the final multivariable logistic regression model, the magnitude of the association between QR and DS/SD was found to be prominent, compared to other covariates (Supplementary Tables S3, S4). QR may be an important factor for depression or insomnia regardless of demographic characteristics and working conditions. Previous study conducted by Kim et al. reported high prevalence of depressive symptoms in Korea (30.7%) and the strongest factor for depression was changes in sleep pattern (9). Therefore, workers with QR, which is highly associated with sleep disturbance, were more likely to be vulnerable to the changes in sleep pattern from the COVID-19 situation. Additionally, interaction analysis found that long working hours and QR had a synergistic interaction with regard to DS and SD based on the synergy index suggested by Rothman et al. (39). This could be one possible explanation for the pronounced association of QR with DS and SD in men (compared to women), since male workers presented a significantly higher prevalence of long working hours and QR (Figure S1). Our study results thus suggest that male workers with long working hours and QR may be considered high-risk of DS and SD. Furthermore, given significantly higher prevalence of SD and DS in women group according to the result of our study as well as previous studies (40, 41), female workers should also be cautiously screened regarding mental health.

Sleep deprivation may be one of the most crucial factors for explaining the association of QR with SD and DS. Several studies have found that QR can shorten sleep duration and cause disrupted and restless sleep (most notably in the shift work system) (42–45). QR may also lead to circadian rhythm disturbances (just like in shift work). SD originating because of QR may form a mediator for the association between QR and depression, as suggested by the mediation analysis results in this study. Considering the plausible mechanism, which is independent of shift work, it may be necessary to conduct further in-depth study on the mechanisms of the QR group among regular workers regarding mental health.

Our study has several strengths. First, the sample utilized in this study was large and encompassed nationwide representative data, which have been frequently validated by other studies. Second, to our knowledge, this study is the first to elucidate the association between QR and depression in workers with various occupation and shift works, while most past studies have emphasized the association of QR with depression in shift workers or health care workers. Third, we employed several statistical methods to examine the association between QR and depression. Moreover, we utilized stratification and interaction analysis to examine vulnerable populations related to QR. However, this study also had several limitations. First, being a cross-sectional study, it could not examine the causal relationship between QR and depression. Further studies with well-designed cohorts should be implemented to clarify the association between QR and risks of developing depression. Second, since this study employed retrospectively collected data, there was a lack of information regarding medical records related to depression and exact frequency of QR occurrence, which could function as unmeasured confounders. Finally, there is a lack of study on the validation of the WHO-5 index among Korean workers, despite its frequent utilization in previous studies (14, 46, 47). Further studies should be conducted to elucidate the validity of WHO-5 index among Korean workers.

In conclusion, our study showed that QR is significantly associated with DS regardless of demographic factors and the working environment. The significant relationship between QR and DS may be mediated by SD. Given the high magnitude of this association presented with high adjusted ORs, workers with QR, in general, should be carefully managed and screened with regards to the risk of developing depression and insomnia. Furthermore, employers and employees both should attempt to keep 11-h uninterrupted recess between work for maintain proper mental health status, accompanied by more detailed policy establishment.

## Data availability statement

The datasets presented in this study can be found in online repositories. The names of the repository/repositories and accession number (s) can be found below: https://oshri.kosha.or.kr/oshri/researchField/workingEnvironmentSurvey.do.

## Ethics statement

The studies involving human participants were reviewed and approved by Severance Hospital's Institutional Review Board. Written informed consent for participation was not required for this study in accordance with the national legislation and the institutional requirements.

## Author contributions

JS and BY wrote the manuscript and were responsible for the conception of the study and data analyses. JO and YK collected data and had processed data. J-HY and BY conceived the ideas. BY, JS, and J-HY contributed with insight, scientific discussion, and editing of the manuscript. All authors contributed to the article and approved the submitted version.

## Funding

This research was supported by a grant of the Korea Health Promotion Institute R&D Project, funded by the Ministry of Health & Welfare, Republic of Korea (grant number: HS21C2367).

## Conflict of interest

The authors declare that the research was conducted in the absence of any commercial or financial relationships that could be construed as a potential conflict of interest.

## Publisher's note

All claims expressed in this article are solely those of the authors and do not necessarily represent those of their affiliated organizations, or those of the publisher, the editors and the reviewers. Any product that may be evaluated in this article, or claim that may be made by its manufacturer, is not guaranteed or endorsed by the publisher.
