# Supplementary material for: The significant association between quick return and depressive symptoms and sleep disturbances in paid workers: A nationwide survey
Source: Front Public Health. 2022 Oct 10;10:990276. doi: 10.3389/fpubh.2022.990276 (PMC9592117; doi:10.3389/fpubh.2022.990276)
Supplement: Supplementary file 1 [file Data_Sheet_1.docx]

**Table S1 Baseline Characteristics of participants stratified by Quick Return in the matched cohort**

| **Variable** | **No Quick Return (n=3582)** | **Quick Return (n=1208)** | **p-value** | **Absolute standardized mean difference** |
| --- | --- | --- | --- | --- |
| **Age** |  |  | 0.023 | 0.075 |
| Mean (SD) | 42.1 (11.0) | 42.9 (10.7) |  |  |
|  |  |  |  |  |
| **Sex** |  |  | 0.651 | 0.019 |
| Women | 580 (16.2%) | 203 (16.8%) |  |  |
| Men | 3002 (83.8%) | 1005 (83.2%) |  |  |
|  |  |  |  |  |
| **Job status** |  |  | 0.87 | 0.017 |
| full-time | 3197 (89.3%) | 1074 (88.9%) |  |  |
| part-time | 294 (8.2%) | 100 (8.3%) |  |  |
| temporary | 91 (2.5%) | 34 (2.8%) |  |  |
|  |  |  |  |  |
| **Working hours** |  |  | 0.618 | 0.007 |
| ≤40 | 1570 (43.8%) | 536 (44.4%) |  |  |
| 41-51 | 1149 (32.1%) | 370 (30.6%) |  |  |
| ≥52 | 863 (24.1%) | 302 (25.0%) |  |  |
|  |  |  |  |  |
| **Working duration** |  |  | 0.48 | 0.018 |
| <1 | 384 (10.7%) | 136 (11.3%) |  |  |
| 1 to <5 | 1263 (35.3%) | 393 (32.5%) |  |  |
| 5 to <10 | 885 (24.7%) | 309 (25.6%) |  |  |
| 10 to <15 | 723 (20.2%) | 262 (21.7%) |  |  |
| ≥15 | 327 (9.1%) | 108 (8.9%) |  |  |
|  |  |  |  |  |
| **Shift Work** |  |  | 0.077 | 0.044 |
| No | 2608 (72.8%) | 847 (70.1%) |  |  |
| Yes | 974 (27.2%) | 361 (29.9%) |  |  |
|  |  |  |  |  |
| **Education** |  |  | 0.025 | 0.033 |
| High school graduate or under | 1165 (32.5%) | 431 (35.7%) |  |  |
| 2-year college | 702 (19.6%) | 199 (16.5%) |  |  |
| Over university | 1715 (47.9%) | 578 (47.8%) |  |  |
|  |  |  |  |  |
| **income (10,000 KRW)** |  |  | 0.377 | 0.033 |
| <200 | 415 (11.6%) | 158 (13.1%) |  |  |
| 200 to <400 | 2275 (63.5%) | 751 (62.2%) |  |  |
| ≥400 | 892 (24.9%) | 299 (24.7%) |  |  |
|  |  |  |  |  |
| **Region** |  |  | 0.569 | 0.017 |
| Rural | 1607 (44.9%) | 554 (45.9%) |  |  |
| Central | 1975 (55.1%) | 654 (54.1%) |  |  |
|  |  |  |  |  |
| **Occupation** |  |  | 0.006 | 0.047 |
| White collar | 1911 (53.3%) | 594 (49.2%) |  |  |
| Blue collar | 1044 (29.1%) | 411 (34.0%) |  |  |
| Others | 627 (17.6%) | 203 (16.8%) |  |  |

**Table S2 Adjusted Odds Ratios (95% CIs) of Depression by Quick Return in Sensitivity Analyses**

|  | **Crude model** | **model 1** | **model 2** | **model 3** |
| --- | --- | --- | --- | --- |
| Cut-off score of ≤28 in WHO-5 Index | 2.24 (1.94-2.59) | 2.31 (2.00-2.66) | 2.44(2.11-2.82) | 2.25 (1.94-2.62) |
| Propensity score matching | 1.97 (1.72-2.25) | 1.96 (1.71-2.24) | 1.95 (1.71-2.23) | 1.97 (1.72-2.25) |
| Participants working more than 1 year | 2.00 (1.77-2.27) | 2.06 (1.82-2.33) | 2.16 (1.90-2.45) | 1.99 (1.75-2.27) |

Model 1: adjusted by age

Model 2: adjusted by age, income, and education

Final model: adjusted by age, income, education, working hours, sleep disturbance, job status, working duration, region, shift work, and occupation

**Table S3 Multivariable logistic models of depressive symptom**

|  | **crude model** | **model 1** | **model 2** | **final model** |
| --- | --- | --- | --- | --- |
| **QR** |  |  |  |  |
| No | 1.00 (reference) | 1.00 (reference) | 1.00 (reference) | 1.00 (reference) |
| Yes | 1.98 (1.77-2.23) | 2.04 (1.81-2.29) | 2.17 (1.93-2.44) | 2.01 (1.78-2.27) |
| **Age** |  | 1.02 (1.01-1.02) | 1.01 (1.01-1.01) | 1.01 (1.01-1.01) |
| **Sex** |  |  |  |  |
| Men |  | 1.00 (reference) | 1.00 (reference) | 1.00 (reference) |
| Women |  | 0.88 (0.83-0.94) | 0.95 (0.89-1.01) | 0.92 (0.86-0.98) |
| **income (10,000 KRW)** |  |  |  |  |
| <200 |  |  | 1.00 (reference) | 1.00 (reference) |
| 200 to <400 |  |  | 0.86 (0.81-0.91) | 0.82 (0.76-0.88) |
| ≥400 |  |  | 0.70 (0.64-0.77) | 0.67 (0.60-0.74) |
| **Education** |  |  |  |  |
| High school graduate or under |  |  | 1.00 (reference) | 1.00 (reference) |
| 2-year college |  |  | 0.83 (0.77-0.90) | 0.89 (0.82-0.96) |
| Over university |  |  | 0.74 (0.69-0.79) | 0.81 (0.75-0.88) |
| **Working hours** |  |  |  |  |
| ≤40 |  |  |  | 1.00 (reference) |
| 41-51 |  |  |  | 1.09 (1.02-1.17) |
| ≥52 |  |  |  | 1.44 (1.31-1.58) |
| **Job status** |  |  |  |  |
| full-time |  |  |  | 1.00 (reference) |
| part-time |  |  |  | 1.00 (0.91-1.09) |
| temporary |  |  |  | 1.51 (1.34-1.71) |
| **Working duration (year)** |  |  |  |  |
| <1 |  |  |  | 1.00 (reference) |
| 1 to <5 |  |  |  | 0.92 (0.85-1.00) |
| 5 to <10 |  |  |  | 0.94 (0.85-1.03) |
| 10 to <15 |  |  |  | 0.98 (0.88-1.08) |
| ≥15 |  |  |  | 0.90 (0.79-1.03) |
| **Region** |  |  |  |  |
| Rural |  |  |  | 1.00 (reference) |
| Central |  |  |  | 1.06 (1.00-1.11) |
| **Shift Work** |  |  |  |  |
| No |  |  |  | 1.00 (reference) |
| Yes |  |  |  | 1.01 (0.92-1.10) |
| **Occupation** |  |  |  |  |
| White collar |  |  |  | 1.00 (reference) |
| Blue collar |  |  |  | 1.10 (1.02-1.19) |
| Others |  |  |  | 0.87 (0.80-0.94) |

**Table S4 Multivariable logistic models of sleep disturbance**

|  | **crude model** | **model 1** | **model 2** | **final model** |
| --- | --- | --- | --- | --- |
| **QR** |  |  |  |  |
| No | 1.00 (reference) | 1.00 (reference) | 1.00 (reference) | 1.00 (reference) |
| Yes | 3.53 (3.15-3.97) | 3.67 (3.26-4.13) | 3.70 (3.28-4.16) | 3.24 (2.87-3.66) |
| **Age** |  | 1.02 (1.02-1.02) | 1.02 (1.02-1.02) | 1.02 (1.02-1.02) |
| **Sex** |  |  |  |  |
| Men |  | 1.00 (reference) | 1.00 (reference) | 1.00 (reference) |
| Women |  | 0.86 (0.80-0.92) | 0.87 (0.81-0.93) | 0.85 (0.80-0.92) |
| **income (10,000 KRW)** |  |  |  |  |
| <200 |  |  | 1.00 (reference) | 1.00 (reference) |
| 200 to <400 |  |  | 0.96 (0.90-1.03) | 0.85 (0.79-0.92) |
| ≥400 |  |  | 0.87 (0.79-0.97) | 0.75 (0.67-0.84) |
| **Education** |  |  |  |  |
| High school graduate or under |  |  | 1.00 (reference) | 1.00 (reference) |
| 2-year college |  |  | 0.93 (0.85-1.01) | 0.93 (0.85-1.01) |
| Over university |  |  | 1.06 (0.99-1.14) | 1.08 (0.99-1.17) |
| **Working hours** |  |  |  |  |
| ≤40 |  |  |  | 1.00 (reference) |
| 41-51 |  |  |  | 1.28 (1.19-1.38) |
| ≥52 |  |  |  | 1.53 (1.38-1.69) |
| **Job status** |  |  |  |  |
| full-time |  |  |  | 1.00 (reference) |
| part-time |  |  |  | 0.89 (0.80-0.98) |
| temporary |  |  |  | 1.23 (1.08-1.41) |
| **Working duration (year)** |  |  |  |  |
| <1 |  |  |  | 1.00 (reference) |
| 1 to <5 |  |  |  | 0.88 (0.80-0.97) |
| 5 to <10 |  |  |  | 0.95 (0.86-1.06) |
| 10 to <15 |  |  |  | 1.08 (0.96-1.20) |
| ≥15 |  |  |  | 0.94 (0.82-1.09) |
| **Region** |  |  |  |  |
| Rural |  |  |  | 1.00 (reference) |
| Central |  |  |  | 1.03 (0.97-1.09) |
| **Shift Work** |  |  |  |  |
| No |  |  |  | 1.00 (reference) |
| Yes |  |  |  | 1.24 (1.12-1.36) |
| **Occupation** |  |  |  |  |
| White collar |  |  |  | 1.00 (reference) |
| Blue collar |  |  |  | 0.89 (0.82-0.97) |
| Others |  |  |  | 0.82 (0.75-0.89) |


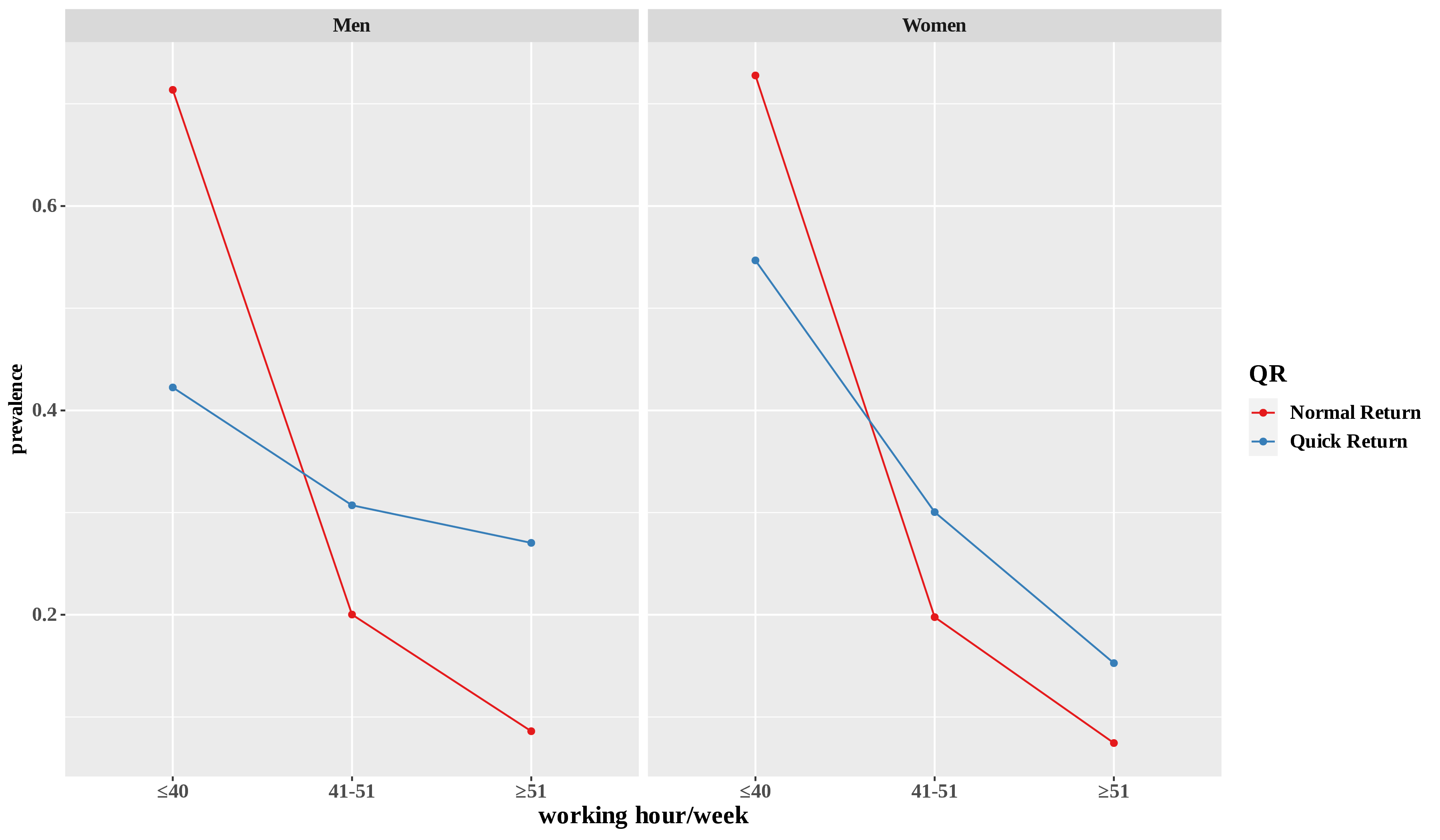


Figure S1 The prevalence of long working hours and quick return stratified by gender
